# Modulation of Visual Working Memory Performance via Different Theta Frequency Stimulations

**DOI:** 10.3390/brainsci11101358

**Published:** 2021-10-15

**Authors:** Xue Guo, Ziyuan Li, Liangyou Zhang, Qiang Liu

**Affiliations:** 1 Institute of Brain and Psychological Sciences, Sichuan Normal University, Chengdu 610066, China; gx@stu.sicnu.edu.cn (X.G.); sccd_zly@126.com (L.Z.); 2 Research Center of Brain and Cognitive Neuroscience, Liaoning Normal University, Dalian 116029, China; 15097977739@163.com

**Keywords:** visual working memory capacity, quality, quantity, tACS

## Abstract

Previous studies have found that transcranial alternating current stimulation (tACS) can significantly enhance individuals’ working memory performance. However, it is still unclear whether the memory performance enhancement was attributed to the quantity or the quality of working memory. The current study applies tACS over the right parietal cortex at slower (4 Hz) and faster (7 Hz) frequencies to participants with high and low working memory capacities in a color recall memory task. This enabled us to explore the tACS effects on the quantity and quality of the working memory for individuals with different memory capacities. The results revealed that slower frequency (4 Hz) tACS enhanced the quality of memory representations, and faster frequency (7 Hz) tACS principally impaired the quantity of working memory. The underlying mechanism of this effect might be that tACS at different frequencies modulate the memory resources, which then selectively affect the quantity and quality of memory representations. Importantly, individual traits, as well as memory strategies, may be crucial factors to consider when testing the effect of tACS on working memory performance.

## 1. Introduction

The visual working memory (VWM) system is responsible for the short-term retention and processing of visual information from the external environment [1,2]. As the core of cognition, VWM is strongly related to a wide range of higher cognitive abilities, such as fluid intelligence, attentional control, and reasoning [3,4,5]. Thus, researchers have devoted much effort to investigating how to improve memory performance in the field of VWM.

Previous studies have confirmed that a certain period of training for working memory can significantly improve working memory performance [6,7,8]; such training, however, is time consuming and restricted by specific conditions. Thus, researchers have been seeking methods to rapidly improve working memory performance. Recently, transcranial alternating current stimulation (tACS), a noninvasive stimulation technique, has been adopted to modulate the neural oscillations in a frequency-specific manner by delivering weak electrical currents to the scalp of the brain [9,10], achieving the goal of memory performance enhancement.

In the field of VWM, tACS is commonly delivered at a specific frequency over the parietal cortex to modulate the theta oscillations, which relate to working memory processes [11,12,13,14,15]. Jaušovec applied tACS to the left parietal cortex and the left frontal cortex at a frequency slower than that of the individual theta during a change detection task. This slower frequency was obtained by adjusting the individual theta frequency (ITF). The ITF was estimated by subtracting a specific value from the individual alpha peak frequency (IAF), which was calculated by collecting the resting state EEG. The results showed that the working memory capacity (WMC) improved when delivering tACS over the parietal cortex only, rather than over the frontal cortex [12]. In addition, previous findings from behavioral and neuroelectric data have revealed a causal relation between working memory storage and theta frequency oscillations in the parietal area [12]. Thus, the theta frequency oscillation in the parietal cortex appears to have a strong correlation with the WMC. Consistently, it was found that tACS effects were observed only for the experimental montage, in which the right parietal electrode was paired with the right supraorbital return electrode, but not for the control montage, in which the Cz return electrode was paired in a previous study [15]. In that study, the tACS effect was tested by stimulating the target regions at slower (4 Hz) and faster (7 Hz) frequencies, compared with the sham tACS in a change detection task. The results showed that the K value (the estimate of the WMC) was significantly improved when stimulated at 4 Hz; on the contrary, the K value significantly decreased when stimulated at 7 Hz [15]. Similar findings were also observed in a study conducted by Bender et al., where the right parietal region was stimulated by tACS at 4 Hz and 7 Hz frequencies. The results showed that the 4 Hz frequency tACS could significantly improve the WMC relative to 7 Hz [11]. Overall, these empirical findings consistently confirmed that tACS influenced the working memory performance by modulating the parietal cortex primarily; and, importantly, the working memory performance could be improved by stimulation at a frequency close to or slower than individual theta oscillation.

The theta–gamma coupling theory can account for these results, explaining why slower theta oscillations can improve the WMC. This theory proposes that the theta and gamma oscillations interact in the same brain region and a gamma cycle represents a single memory item [16,17,18]; the gamma cycles could be nested into a theta wave and the length of a theta wave could determine the number of gamma cycles nested into one theta wave. In other words, more gamma cycles could be nested into a theta wave when the theta wave was slower, which meant that more memory items could be retained in the VWM, corresponding to a higher WMC.

Recent research has proposed that the storage capacity of working memory might not be a unitary construct, and there may be a distinction between the number of items stored in the VWM and the precision or resolution of those memory representations [19]. Indeed, it was found that there was no correlation between the estimated values of number and precision. In other words, individuals who could retain the largest number of items in their working memory capacity were not necessarily the same individuals who had the clearest memories [20,21]. In the current study, we selected quantity and quality to refer to the number and precision of items held in the VWM, respectively. In the two-factor model, the quantity and quality represent distinct facets of working memory ability, which jointly determine the VWM performance. Regarding the previous results obtained from the change detection tasks, we failed to discern whether the improvement of VWM performance benefited from quantity or quality, after the application of tACS. Therefore, it is still unclear whether the improvement of memory performance by low-frequency theta stimulation was attributed to the quantity or quality of memory representations. Thus, this study aims to explore this issue.

To concurrently index the quantity and quality of working memory, the current study adopted a color recall memory task [22]. We attempted to ascertain how tACS affected the quantity and quality of VWM by delivering tACS over the right parietal cortex. Participants with different WMCs indeed had different theta peak frequencies and, conversely, the theta frequency was strongly related to individual WMCs [23]. Given that the intrinsic theta wave ranged in frequency between 4 Hz and 7 Hz, we selected the 4 Hz and 7 Hz tACS to modulate individual parietal theta frequencies, which allowed us to effectively observe the tACS effect on the quantity and quality of VWM. According to the theta–gamma coupling theory, which proposes that theta frequency can determine individual WMCs, it could be considered that the theta frequency of the high WMC group was close to 4 Hz, while the low WMC group was close to 7 Hz. Notably, previous studies have found that when multiple items needed to be encoded in memory tasks, participants with a low WMC strategically adopted an encoding-all manner, regardless of whether they could remember these items. Conversely, those with a high WMC tended to adopt an encoding-partial manner to ensure that some items would definitely be remembered [24,25,26]. Therefore, the test implied that there was an intricate interaction between tACS and individual differences. Thus, participants were assigned to a high WMC or low WMC group in the current study, so that we could explore the tACS effect on the quantity and quality of VWM for people with different WMCs.

## 2. Materials and Methods

### 2.1. Participants Screening for WMC

All participants were pre-screened with a partial report change detection task [27]. In this task, participants were instructed to determine whether the color of probe items was identical to that of the memory items. Six colored squares were presented for memorization in each trial. To acquire a sufficient sample size, we collected data on a large scale on campus using the mobile version of the WeChat applet. The K value of each participant was obtained, which was the standardized estimate of the WMC that took the set size (i.e., the number of items presented in each memory array) into account. The K value was calculated using the formula, K = S * (H − F), where H is the hit rate, F is the false alarm rate, and S is the set size [28]. H is the hit rate which denotes a “yes” answer to targets and F is the false alarm rate which denotes a “yes” answer to non-targets. A correlation analysis of memory capacity was conducted between tests of the mobile version and the computer version (r = 0.72, *p* < 0.001). A total sample size of 207 was collected in the screening experiment; the average K value was 2.36. Due to the large attrition of participants, we customized the grouping criteria for the high and low WMCs. Participants with K values lower than 2.5 were classified as belonging to the low WMC group, while those with K values higher than 3 were placed in the high WMC group. Finally, we informed participants who were willing to participate in the subsequent formal experiment to arrive at the laboratory by appointment.

### 2.2. Participants

Based on the power analysis of a previous experiment [15], a medium effect size f = 0.25 with an alpha level of 0.05 and a statistical test power of 0.95 were selected. The total sample size obtained by prior analysis was at least 28. Participants were recruited from Sichuan Normal University. In this experiment, 44 healthy right-handed college students (14 males, ranging in age from 18 to 25 years, with a mean age of 20.94 years) voluntarily participated. They had a similar education level without an obvious intellectual deviation. They reported normal vision or corrected-to-normal vision and normal color vision. None of the participants reported a diagnosis of neurological or psychiatric disorder. They signed the informed consent before participation in the experiment and received corresponding compensation after completion. The study was conducted according to the guidelines of the Declaration of Helsinki and approved by the Ethics Committee of the Institute of Brain and Psychological Sciences, Sichuan Normal University.

### 2.3. Color Recall Memory Task

**Figure 1:**
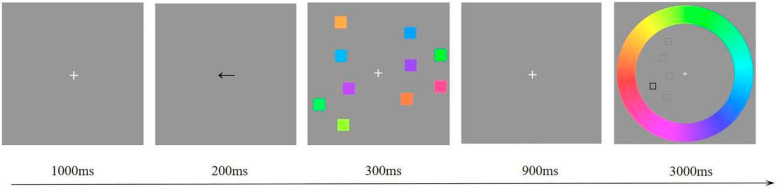
A schematic illustration of the color recall memory task.

The memory stimuli were presented on a 23.8 inch screen (1920 × 1080 resolution and at a 60 Hz refresh rate). The screen background was gray and the luminance was 150°. As Figure 1 depicts, five memory items were presented on each side of the fixation cross. The color of each memory item (0.8° × 0.8°) was randomly selected from a uniformly distributed 360° color ring. The color difference between any two squares was larger than 30°, and the distance between the centers of any two squares was not less than 2°. A color ring was presented in a probe array. The outer and inner diameters of the color ring were 8.2° and 6°, respectively. The color ring constantly scrolled between trials throughout the experiment. In the color ring, the hollow squares were presented as spatially compatible with the positions of the memory items; the target item was peripherally bolded. Participants performed the experiment at a viewing distance of 60 cm in a softly lit and comfortable chamber.

Each trial started with a fixation cross presented in the center of the screen for 1000 ms. Then, an arrow was presented to randomly point left or right for 200 ms, indicating which side presented memory items. Five memory items on each side of the memory array were kept visible for 300 ms. Followed by a retention interval of 900 ms, the probe array presented a color ring in which five hollow squares in the cued side were located in the same positions as those of the memory array, and the target item was peripherally bolded. Participants were asked to retrieve the target color and select it by clicking on the color ring with the mouse within 3000 ms. The color and positions of memory stimuli were randomly assigned by a computer program without replacement in a trial. The experiment was divided into three different phases, each consisting of 300 trials.

### 2.4. tACS Protocol

**Figure 2:**
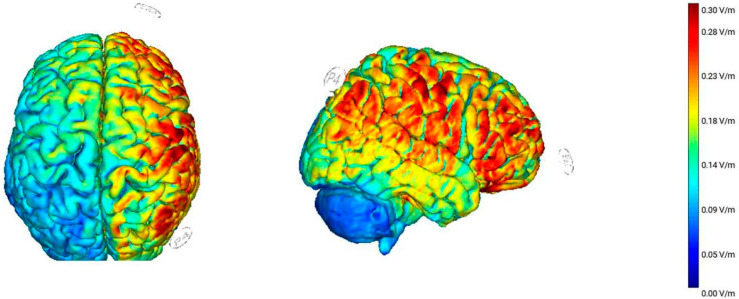
The tACS electrode configuration and cortical activation induced by current simulation. The tACS sponge electrodes are placed on the right parietal cortex (P4) and the right eyebrow orbit (FP2). Using the software NIC2.0, the norm of the electric field (V/m) is depicted on an example brain from a transverse and right sagittal view.

StarStim8 (Neuroelectrics, Barcelona, Spain) was used to deliver the tACS. Prior to the electrode placement, the scalp and skin were wiped with an alcohol pad to improve electrical conductivity. Two 5 × 5 cm sponge electrodes were soaked in saline. Then, one was placed on the right parietal cortex (P4) and the other was placed on the right eyebrow orbit (FP2) as reference. The tACS electrode configuration and cortical activation induced by electrical simulation were shown in Figure 2. After the placement of the electrodes, we started to modulate the current threshold for each participant. The maximum stimulation intensity was initially set to 1500 μA of peak-to-peak. In the first phase, stimulation at 1500 μA was tested to see if retinal phosphenes or other tingling sensations occurred. If none occurred, this level of stimulation was used. Otherwise, the threshold was determined by starting at 1000 μA and increased by increments of 100 μA or decreased by increments of 50 μA, until the highest level that did not produce retinal phosphenes or other sensations was found. In the second and third phases, the current was correspondingly adjusted according to the current threshold determined in the first stage. There were three types of stimulation frequencies: 4 Hz, 7 Hz, and sham stimulation. In the 4 Hz and 7 Hz stimulation conditions, stimulation could be achieved at the threshold of 1500 μA. A brief stimulation period was performed in the sham stimulation condition to mimic any sensations that might be felt in the active stimulation conditions. The mean stimulation level for all participants was 1090 μA. Participants reported experiencing the sensation of retinal phosphenes when using the previously determined stimulation of 7 Hz, thus resulting in a mean reduction of 140 μA during the 7 Hz stimulation compared to the 4 Hz stimulation. During the ramp up, stimulation, and ramp down of the sham stimulation, the stimulation frequency was set at 5.5 Hz, which was selected as the neutral frequency relative to the two active stimulation conditions. The ramp-up and ramp-down periods of each stage were 30 s. During the sham stimulation, participants performed the task only after the ramp-down period. The impedance level was maintained below 10 kΩ during all stimulations.

After the adjustment of the current threshold, participants were instructed to respond with the mouse. They had a short practice session before the formal experiment; feedback was provided during the practice phase. After the practice phase, additional saline was added to the sponge and the tACS stimulation began. Participants were then instructed to begin the experiment when they were ready. The total task time during the stimulation was about 20 min. To avoid interference between the effects of the tACS at different frequencies, participants received the 4 Hz, 7 Hz, and sham tACS settings on three separate days, scheduled at least 24 h apart. They were asked to come to the lab at approximately the same time-point to ensure that they were in the same mental state during each stimulation. A within-subjects design was used for stimulation protocol. The 4 Hz, 7 Hz, and sham tACS settings were delivered in different sessions, and the order of the tACS sessions was counterbalanced across participants. The experiment was conducted in a single-blind design, with participants unaware of which stimulation was administered at any given stage.

### 2.5. Data Analysis

The color recall paradigm subtracted the value of the target color in the color space from the value of the reported color, to calculate the error degree in each trial in the experiment. Then Memtoolbox [29] was used to analyze the error degree. The standard mixed model was used to fit the data to obtain three independent values to independently measure the M, sd, and g values [22]. The M value reflected the system deviation, and no obvious system deviation was found in this experiment. The sd value represented the width of the error distribution of the reported color encoded in the memory, which was opposed to the precision of representations, so quality (precision) could be characterized by the reciprocal of sd. The g value represented the guess rate and was used as an indicator of working memory quantity (1-g). The guess rate meant that participants randomly responded to the probe color due to the probe item not being encoded into the working memory system. Here, we also used the K value to denote the WM quantity, and it was calculated by a set size (1 − g)*.

## 3. Results

**Figure 3:**
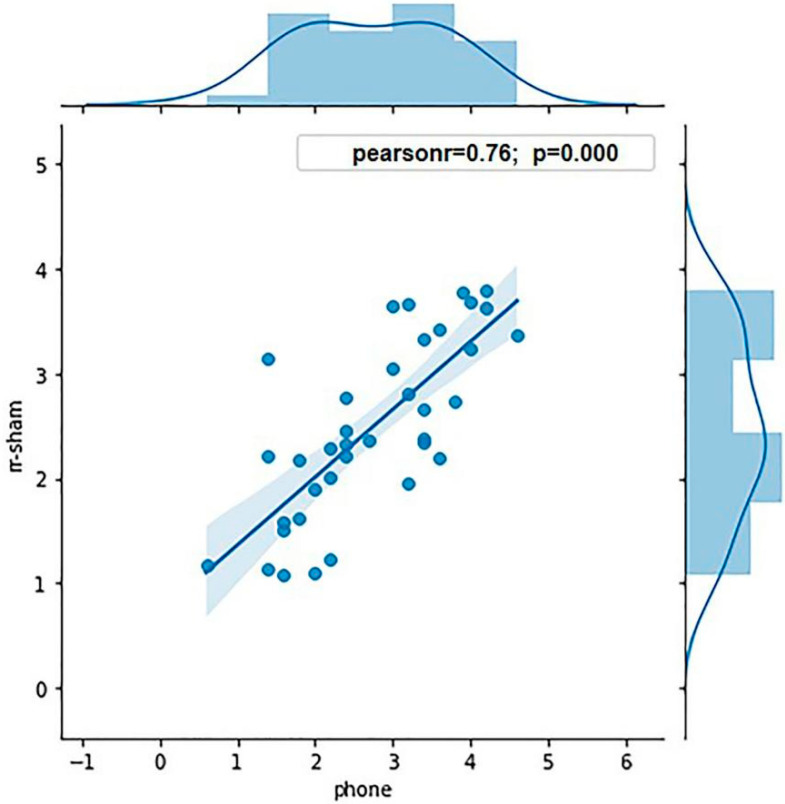
The correlation of the K value measured by the mobile phone and in the sham stimulation condition in this experiment.

Eight participants were excluded from the experiment due to their sd value being three times greater than the mean sd value, and the g value being greater than 0.8. Thus, the data of 36 participants were analyzed: 18 participants in each high and low WMC group. The average K values of the high and low WMC groups were 3.62 and 1.86, respectively. ANOVA was conducted to analyze the WMCs of the two groups, showing a significant effect of grouping (*p* < 0.001). To ensure the validity of grouping in terms of the WMC, the Pearson correlation analysis was conducted between the K value calculated in the sham stimulation condition and measured by the mobile phone (r = 0.76, *p* < 0.001; Figure 3), showing that the grouping was predictive.

A mixed factorial ANOVA with the between-factor group (the high WMC group and the low WMC group) and the within-factor stimulation type (4 Hz, 7 Hz, and sham) × hemifield (left hemifield and right hemifield) was carried out on the g value and sd value. For the g value, the results showed a main effect of hemifield, F(1,34) = 14.446, *p* = 0.001, η^2^ = 0.298. The interaction between the stimulation type and the group was significant (F(2,68) = 5.256, *p* = 0.008, η^2^ = 0.134), indicating that the two groups were modulated differently in memory performance depending on the stimulation type. For the sd value, the main effect of the stimulation type was significant (F(2,68) = 3.251, *p* = 0.045, η^2^ = 0.087), and the interaction between the stimulation type and the group was also significant (F(2,68) = 3.662, *p* = 0.031, η^2^ = 0.097). Subsequent ANOVAs with stimulation type and hemifield were then performed separately for the high and low WMC groups.

### 3.1. High WMC Group

**Figure 4:**
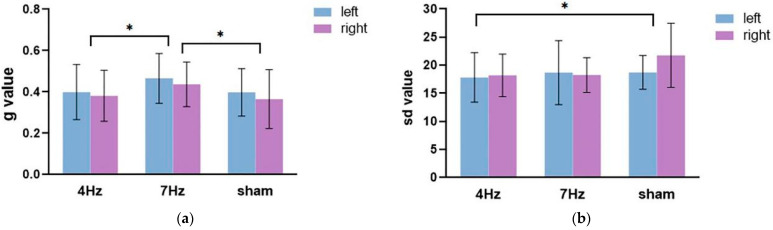
The results of the guess rate (**a**) and precision (**b**) in the three stimulation conditions (4 Hz/7 Hz/sham) for the high WMC group. The blue bar represents the left hemifield and the purple bar represents the right hemifield. Note that higher sd values imply a worse quality and higher g values mean a lower quantity of working memory. The asterisks (*) at the top of bar graph denote *p* < 0.05.

The repeated-measures ANOVA on the guess rate (Figure 4a) showed that the main effect of the stimulation type was significant (F(2,34) = 6.673, *p* = 0.004, η^2^ = 0.282), and the main effect of the hemifield was significant (F(1,17) = 5.970, *p* = 0.026, η^2^ = 0.260). The interaction between the hemifield and stimulation type was not significant (F(2,34) = 0.129, *p* = 0.879, η^2^ = 0.008). Pairwise comparison analysis revealed that the guess rate of working memory was higher in the left hemifield than the right hemifield; the guess rate at a stimulation of 7 Hz was significantly higher than that at a stimulation of 4 Hz (*p* = 0.009) and a sham stimulation (*p* = 0.003). These results suggested that the quantity of working memory was significantly lower in the 7 Hz stimulation relative to the sham and 4 Hz stimulations.

The repeated-measures ANOVA for precision results (Figure 4b) showed that the main effect of stimulation was significant (F(2,34) = 3.466, *p* = 0.043, η^2^ = 0.169). The main effect of the hemifield was not significant (F(1,17) = 1.521, *p* = 0.234, η^2^ = 0.082). The interaction between the two factors was also not significant (F(2,34) = 1.390, *p* = 0.263, η^2^ = 0.076). Pairwise comparison analysis showed that the quality at 4 Hz stimulation was significantly higher and relative to the sham stimulation (*p* = 0.023).

### 3.2. Low WMC Group

**Figure 5:**
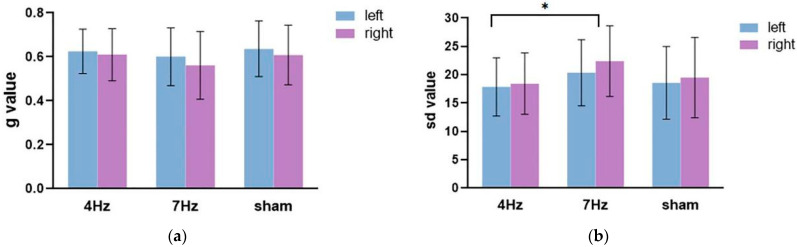
The results of the guess rate (**a**) and precision (**b**) in the three stimulation conditions (4 Hz/7 Hz/sham) for the low WMC group. The blue bar represents the left hemifield and the purple bar represents the right hemifield. Note that higher sd values imply a worse quality and higher g values mean a lower quantity of working memory.The asterisks (*) at the top of bar graph denote *p* < 0.05.

The repeated-measures ANOVA on the guess rate (Figure 5a) showed that the main effect of the hemifield was significant, F(1,17) = 8.991, *p* = 0.008, η^2^ = 0.346. The main effect of stimulation was not significant (F(2,34) = 1.068, *p* = 0.355, η^2^ = 0.059), nor was there a significant interaction between the hemifield and stimulation (F(2,34) = 0.271, *p* = 0.764, η^2^ = 0.016). The following paired test showed that the guess rate in the left hemifield was significantly higher than in the right hemifield.

The repeated-measures ANOVA on the precision (Figure 5b) showed that the main effect of the hemifield was not significant, F(1,17) = 1.444, *p* = 0.246, η^2^ = 0.078. The main effect of stimulation was significant (F(2,34) = 5.669, *p* = 0.014, η^2^ = 0.415), and the interaction between the hemifield and stimulation type was not significant (F(2,34) = 0.117, *p* = 0.891, η^2^ = 0.014). The results of the pairwise comparison analysis showed that the quality at 4 Hz stimulation was significantly enhanced, relative to the stimulation at 7 Hz (*p* = 0.004). Although the quality difference between tACS at 4 Hz and sham stimulation failed to approach any significance, the results revealed that the low WMC group tended to exhibit a better performance at 4 Hz stimulation relative to sham stimulation.

## 4. Discussion

The current study aimed to explore how electrical stimulation affected working memory performance by applying tACS at 4 Hz and 7 Hz frequencies over the right parietal region, during a traditional color recall memory task, in which g and sd values were used to index the quantity and quality of memory representations, respectively. Considering individual differences in theta oscillations, participants were assigned to high or low WMC groups. These results showed that for the high WMC group, 4 Hz tACS can improve the quality of working memory relative to sham stimulation, whereas 7 Hz tACS relative to sham stimulation greatly reduced the quantity of working memory. For the low WMC group, active tACS had little effect on the quantity of working memory, while 4 Hz tACS significantly enhanced the quality of working memory relative to the stimulation at 7 Hz. Additionally, the quality under the 4 Hz stimulation exhibited a boost trend relative to the sham stimulation. Overall, in line with the study of Wolinski et al. [15], these results indicated that a 4 Hz theta frequency can indeed enhance working memory performance, while 7 Hz theta frequency impaired memory performance. Importantly, in light of the results indicating that not all participants showed a consistent effect of tACS on the quantity and quality of working memory, the current study offers a new presumption that the tACS’s modulation of memory performance can be very different in the high WMC group compared to the low WMC group.

According to the stipulations of the theta–gamma coupling theory, which proposes that more gamma cycles can be nested into a theta wave if theta oscillations are slower, we deduced that stimulation at 4 Hz relative to 7 Hz would boost the quantity of working memory, regardless of an individual’s WMC. However, this theory did not perfectly match the current results, which suggested that the tACS effect on the quantity of working memory only occurred in the high WMC group. Unlike the theta–gamma coupling theory, the slot + resources model proposed that an individual possessed roughly 3–4 slots and each slot represented a single memory item. The slots, regarded as memory resources, could be flexibly allocated among these memory representations, which meant that multiple slots could be used to represent one memory item to improve the quality of that memory representation [22]. Thus, if the number of gamma cycles was conceptually equivalent to the number of slots, then the improvement of memory quantity and/or quality could be attributed to the increase in slots. Therefore, there might be more slots under the 4 Hz stimulation relative to the 7 Hz stimulation regardless of the WMC. This deduction, however, failed to account for why tACS had different effects on working memory performance between the low and high WMC groups. We might plausibly interpret the current results by taking the encoding strategy into consideration.

Specifically, participants could determine the optimal number of items they could remember during the practice stage, according to their WMC and the set size of memory array, and then continuously used this strategy to perform the remaining trials. As a result, the theta frequency of the high WMC group was presumably close to 4 Hz, they possessed relatively more slots, and could thus retain more memory items. Considering that individuals would encode a fixed number of memory items throughout the whole task and 4 Hz stimulation slightly increased the number of memory slots, the enhancement of memory resources by 4 Hz stimulation was possibly assigned to improve the quality of memory representations. In contrast, stimulation at 7 Hz could significantly reduce the number of memory slots, resulting in an insufficient number of slots in the condition that the participants continuously kept relatively more memory items; thus, the quantity of working memory showed a significant decrease in the 7 Hz stimulation condition relative to the sham stimulation. This deduction also was in line with the results from the low WMC group. It was supposed that the theta frequency of an individual with a low WMC was close to 7 Hz and relatively fewer items were retained. Then, 4 Hz stimulation could significantly increase the number of memory slots, a while 7 Hz stimulation could slightly decrease the number of memory slots. As the low WMC group was likely to constantly encode a small number of items, the quantity of working memory was comparable, regardless of whether the memory slots had increased. In contrast, the quality of working memory benefited from a 4 Hz stimulation, which produced more slots relative to a 7 Hz tACS and sham stimulation. In brief, it can be concluded that a 4 Hz frequency tACS can boost the quality of working memory, while a 7 Hz frequency tACS principally impaired the quantity of working memory, which, to some extent, was consistent with the theta–gamma coupling theory. Importantly, it should be noted that the tACS effect was modulated by individual traits as well as memory strategies. In future stimulation protocols, it is necessary to consider the impact of these factors on the tACS effect to further optimize the stimulation protocols.

The theta–gamma coupling theory proposed that a gamma cycle was thought to represent a memory item retained in the VWM; thus, the number of gamma cycles (or memory items) increased as the theta frequency became slower in the context of the individual gamma frequency being kept at a fixed value. By the same logic, we might possibly observe the effect of tACS on the frequencies ranging in gamma oscillation on working memory performance. In light of our interpretation of the current results, by combining the theta–gamma coupling theory and the slot + resources model, the gamma cycles were conceptualized as the slots, which were also regarded as a form of resource. When we applied the tACS at a frequency faster than the individual gamma frequency, more gamma cycles could be nested into a theta wave, producing more slot resources; thus, the tACS, at a faster gamma frequency, could possibly facilitate the working memory performance. In contrast, tACS at a frequency lower than the individual gamma frequency would reduce the number of slots, resulting in the impairment of memory performance due to the decreased resources. The current presumption was compatible with the findings from the research of Alekseichuk et al., which observed the enhancement of working memory performance when there was a burst of high gamma oscillations over theta waves [30]. In future research, we could also consider applying different theta frequencies stimulation with a constant gamma stimulation, and the results would be interesting. These presumptions seem to reasonably explain the relationship between theta–gamma coupling and the working memory performance which, nevertheless, is necessarily verified in further in this study.

Regarding the noninvasive stimulation of the brain cortex, the neural activation may not merely be confined to the parietal area when electrical stimulation is applied to that cortex, but, instead, the adjacent brain areas (e.g., frontal areas) can also be activated. Consistent with the current E-field activation map, we observed that the application of tACS over the parietal cortex in the right hemisphere induced neural activity that largely spread from the parietal cortex to the frontal regions. Therefore, whether the tACS effect was specific to the parietal cortex could not be certainly ascertained. Notably, it was proven that theta activity in the parietal cortex was strongly related to the VWM in a previous study, and there was a negligible role of frontal regions in the tACS effect on the VWM performance [12,13]. Thus, the tACS effect could be observed when the parietal cortex was stimulated by tACS at the specific frequencies, indicating the exclusivity of the parietal cortex in the tACS effect on the VWM performance. However, it might not necessarily be confirmed that the tACS effect was solely specific to the parietal cortex. Importantly, the theta frequencies in the parietal cortex indeed strongly correlated with the tACS effect on VWM performance.

Considering the lateralized application of tACS over the scalp, the effect of electrode configuration should be considered. In previous studies, we found a better quantity of working memory in the right hemifield, relative to the left visual hemifield, when applying tACS over the right hemisphere [31,32]. Similarly, applying tACS over the left hemisphere can lead to a greater enhancement of memory quantity in the left visual hemifield [33]. The lateralized effect was also observed in the current study, which showed that the quantity of memory representations in the right (ipsilateral) hemifield was higher than that in the left (contralateral) hemifield, even under the sham stimulation. This might be because the stimulation electrode was placed in either the left or right hemisphere, which possibly caused attention bias to the ipsilateral visual hemifield, and then produced a greater enhancement of memory quantity. Thus, it was plausibly observed that there was a memory advantage to the memory representations in the ipsilateral visual hemifield relative to the position of the stimulation electrode, demonstrating that the electrode position played a relevant role in memory performance.

## 5. Conclusions

To conclude, the current study investigated the tACS effect on the quality and quantity of working memory by applying tACS at the frequencies of 4 Hz and 7 Hz over the right parietal cortex. It has been confirmed that the tACS can indeed modulate the memory resources by modulating the theta frequencies of individuals, thus supporting the theta–gamma coupling theory. In conclusion, the increased resources by tACS at a frequency (4 Hz) slower than individuals’ theta frequencies improved the quality of memory representations, whereas the decreased resources by tACS at a frequency (7 Hz) faster than the individuals’ theta frequencies resulted in the impairment of memory quantity.

## Data Availability

Data are available on request due to restrictions (for example, privacy or ethical). The data presented in this study are available on request from the corresponding authors.
